# Extracellular vesicle-mediated phenotype switching in malignant and non-malignant colon cells

**DOI:** 10.1186/s12885-015-1568-3

**Published:** 2015-08-01

**Authors:** Hillary E. Mulvey, Audrey Chang, Jason Adler, Michael Del Tatto, Kimberly Perez, Peter J. Quesenberry, Devasis Chatterjee

**Affiliations:** Department of Medicine, Rhode Island Hospital and The Alpert Medical School of Brown University, Coro West, Suite 5.01, One Hoppin St, Providence, RI 02903 USA

**Keywords:** Extracellular vesicles, Liquid chromatography-tandem mass spectrometry, Colorectal cancer, Proteomics, 14-3-3, STAT1, IQGAP1, Raf-1, prohibitin, NF-κB, Luciferase reporter assay

## Abstract

**Background:**

Extracellular vesicles (EVs) are secreted from many cells, carrying cargoes including proteins and nucleic acids. Research has shown that EVs play a role in a variety of biological processes including immunity, bone formation and recently they have been implicated in promotion of a metastatic phenotype.

**Methods:**

EVs were isolated from HCT116 colon cancer cells, 1459 non-malignant colon fibroblast cells, and tumor and normal colon tissue from a patient sample. Co-cultures were performed with 1459 cells and malignant vesicles, as well as HCT116 cells and non-malignant vesicles. Malignant phenotype was measured using soft agar colony formation assay. Co-cultures were also analyzed for protein levels using mass spectrometry. The importance of 14-3-3 zeta/delta in transfer of malignant phenotype was explored using siRNA. Additionally, luciferase reporter assay was used to measure the transcriptional activity of NF-κB.

**Results:**

This study demonstrates the ability of EVs derived from malignant colon cancer cell line and malignant patient tissue to induce the malignant phenotype in non-malignant colon cells. Similarly, EVs derived from non-malignant colon cell lines and normal patient tissue reversed the malignant phenotype of HCT116 cells. Cells expressing an EV-induced malignant phenotype showed increased transcriptional activity of NF-κB which was inhibited by the NF--κB inhibitor, BAY117082. We also demonstrate that knock down of 14-3-3 zeta/delta reduced anchorage-independent growth of HCT116 cells and 1459 cells co-cultured with HCT derived EVs.

**Conclusions:**

Evidence of EV-mediated induction of malignant phenotype, and reversal of malignant phenotype, provides rational basis for further study of the role of EVs in tumorigenesis. Identification of 14-3-3 zeta/delta as up-regulated in malignancy suggests its potential as a putative drug target for the treatment of colorectal cancer.

## Background

Colorectal (CRC) cancer is the 2^nd^ most prevalent cancer in women and the 3^rd^ most prevalent cancer among men, globally [1]. Incidence of colorectal cancer is high, with an estimated 1.35 million new cases reported in 2012 [1]. It is estimated that nearly 700,000 patients died of colorectal cancer in 2012 [1]. Recent declines in mortality can be attributed to improved screening processes and treatment plans including surgical resection of tumor tissue, adjuvant chemotherapy and radiation. Postoperative drug therapy is key in targeting micro-metastasis that may be missed during surgery [2]. However, even with medical advances, the average 5-year survival rate of patients is a moderate 64.9 % [3]. This statistic fails to emphasize the impact of disease presentation at diagnosis on the outcome. Early diagnosis of localized disease (stage II) yields a 90.3 % survival rate, but this decreases significantly to a modest 70.4 % survival rate in patients diagnosed with regional disease (stage III). The 20 % of colorectal cancer cases that are diagnosed in late stage, distant disease see only a 12.3 % survival rate (stage IV) [3]. Recent research focuses on the genetic and epigenetic differences in the tumors of colorectal patients to help develop more targeted therapies, tailored to each patient to maximize efficacy. For example, anti-EGFR treatments, including panitumumab and cetuximab, have been shown to improve outcome for patients with tumors carrying wild-type KRAS gene. Conversely, mutations in KRAS, as well as PTEN, BRAF and PIK3CA have been implicated as causative in patients resistant to anti-EGFR treatments [4]. Thus, there is a need for a more expansive body of targeted therapies for improved clinical outcome. Considerable research has focused on understanding the tumor microenvironment, specifically, soluble factors secreted and taken up by cells [5–7]. A variety of cell types release microvesicles (MVs), or exosomes, collectively termed extracellular vesicles. Ranging in size from 30-1000 nm, these vesicles are released into the surrounding environment through fusion of a multivesicular endosome with the plasma membrane or through direct blebbing off the plasma membrane [8]. Once regarded as cellular debris and first studied as mediators of immune action, vesicle contents have been identified to include various types of proteins, nucleic acids and other bioactive molecules. The role of EVs is especially important in regards to tumor progression, because cancer cells have been shown to secrete EVs at an elevated rate. More importantly, higher levels of EVs have been shown to correlate with advanced stages of cancer [9, 10]. Further understanding of the mechanisms underlying increased EV secretion in cancer, and the mechanisms behind phenotypic transfer, are crucial to the potential identification of many novel therapeutic targets.

Recent evidence suggests a role of horizontal transfer of information via EVs in tumorigenesis, including the transfer of specific aspects of cellular phenotype and activation of specific signaling pathways [11]. One study analyzing colorectal cancer EV contents found mutant KRAS protein was present in EVs isolated from DKO-1 cells with KRAS mutation. They were further able to show transfer of the mutant KRAS protein to DKO-8 cells inherently expressing only wild type KRAS, after co-culture with DKO-1 extracellular vesicles [12]. Another study compared protein contents in vesicles isolated from primary CRC cells (SW480) and metastatic CRC cells (SW620). Findings showed differential protein expression, with primary cancer vesicles dense in cell-adhesion proteins and metastatic cancer cells dense in proteins important for regulation of cell cycle [13].

In this study, we demonstrate that EVs play an important role in tumor microenvironment through the promotion of malignant phenotype. We show an induction of malignant phenotype, as measured by growth in soft agar. Anchorage independent growth, a characteristic of malignancy, was induced in non-malignant colon fibroblast cells (1459) after co-culture with EVs isolated from malignant colon cells (HCT116). The reciprocal result occurred with HCT116 cells co-cultured with 1459 vesicles showing a reduction in anchorage independent growth. Vesicles harvested from normal (Colo and 004CT Normal) and malignant (004CT Tumor) patient colon tissue were shown to induce the same changes in phenotype as vesicles isolated from immortalized cell lines, providing rational basis for further exploration into the role of EVs in promotion of metastatic disease. We found an increased expression of the proteins 14-3-3 zeta/delta, prohibitin, phosphorylated Raf kinase Inhibitor Protein (pRKIP) and Signal Transducer and Activator of Transcription1 (STAT1) in 1459 cells acquiring malignant phenotype after co-culture with malignant EVs. Additionally, an increase in transcription of NF-κB was observed after co-culture with malignant EVs. Our study also shows that 14-3-3 zeta/delta is important in the EV mediated induction of malignant phenotype, suggesting it’s role as a potential target for therapeutic interventions.

## Methods

### Materials

All chemicals and reagents were purchased from Sigma Chemical Co. (St. Louis, MO) unless otherwise noted. Protein quantification reagents were obtained from Bio-Rad Laboratories, Inc. (Hercules, CA). Enhanced chemiluminescence reagents as well as secondary mouse and rabbit horseradish peroxidase-conjugated antibodies for Western blot analysis were from GE Healthcare (Arlington Heights, IL). The primary antibodies to Raf-1 (sc-133), actin (sc-1616), prohibitin (sc-6705), STAT1 (sc-346), pRKIP (sc-32623) and 14-3-3 zeta (sc-1019) and 14-3-3 zeta siRNA (sc-29583) were all purchased from Santa Cruz Biotechnology (Santa Cruz, CA). The antibody to RKIP (07-137) was purchased from Millipore (Billerica, MA).

### Cells

Human colon tumor cells were grown in RPMI with 10 % fetal bovine serum, 100 IU/mL penicillin, 100 μg/mL streptomycin, 1 μg/mL metronidazole, 2.5 μg/mL amphotericin and 20 μg/mL gentamicin. The human adenocarcinoma cell line, HCT116 and human normal colon fibroblast cell line 1459 were purchased form ATCC (Rockville, MD). HCT116 cells were cultured in McCoy’s 5A and 1459 cells in Eagle’s Minimum Essential Medium (EMEM). Both mediums were supplemented with 10 % fetal bovine serum (FBS), 1 % glutamine, 1 % non-essential amino acids, 100 IU/mL penicillin and 50 IU/mL streptomycin. All cells were cultured in a humidified incubator containing 5 % CO_2_ at 37 °C.

### Western blot analysis

Total cell extracts were prepared as previously stated [14]. The protein concentrations of lysates were determined using either BCA protein assay kit (Pierce) or Bradford assay kit (BioRad). Proteins were separated using 10 % SDS-PAGE and electrophoretically transferred from the gel to nitrocellulose membranes (GE Healthcare). Enhanced chemiluminescence reagents (GE Healthcare) were used to detect proteins recognized by the antibodies.

### Tissue collection

Human colon tissue samples were obtained on October 23, 2012 and January 11, 2013. Consent for the collection of patient human colon cancer samples was written and informed and obtained according to the Rhode Island Hospital’s Committee on Protection of Human Subjects (Institutional Review Board). The tumor samples were collected and transported in Roswell Park Memorial Institute medium (RPMI) supplemented with 500 IU/mL penicillin, 500 mg/mL streptomycin, 100 μg/mL gentamicin, 12.5 μg/mL amphotericin and 5 μg/mL metronidazole.

### Extracellular vesicle isolation

Extracellular vesicles were isolated from two different patient tissue samples under approval of the Institutional Review Board at Rhode Island Hospital. Tissue samples were weighed and minced with a sterile scalpel into 1-2 cm pieces as previously reported [15]. Enzymatic dissociation was performed on each of the tissue pieces using 0.2 % collagenase in RPMI with 10 % FBS for 90 min at 37 °C. The tissue pieces were passed sequentially through 18, 22 and 25 gauge needles followed by a 40 um cell strainer, as reported [15]. Resulting cell suspensions were washed twice with RPMI and grown in a T-75 tissue culture flask. Cells were grown in RPMI media supplemented with 10 % EV-free FBS, 1 % glutamine, 1 % nonessential amino acids, 100 IU/mL penicillin and 50 IU/mL streptomycin. HCT116 cells and 1459 cells were plated at 1.5x10^5^cells per T-75 flask prior to vesicle isolation.

Conditioned medium was collected 7 days after culture and processed to isolate the extracellular vesicles. The medium was centrifuged at 300 x gravity for 10 min at 4 °C. The UCF (ultra centrifuged) supernatant was isolated after centrifugation at 28,000 x gravity for 1 h at 4 °C. The UCF pellet was suspended in growth medium and co-cultured with cells for 7 days (see below).

### Protein lysate preparation from EVs

EVs were harvested from HCT116 cells and underwent differential centrifugation: 300 x g for 10 min, 2000 x g for 30 min, 10,000 x g for 30 min (microvesicle pellet) and then 100,000 x g for 60 min (exosome pellet). To isolate the total EV fraction, the 10,000 x g was skipped after the 2000 x g spin. The different fractions were lysed in RIPA lysis buffer for 30 min on ice. The supernatant was collected for Western blot analysis following centrifugation at 13,000 rpm for 10 min. For Western blot analysis, 10 μg protein samples were separated on 10 % SDS-PAGE and transferred to a nitrocellulose membrane.

### Co-culture of colon derived extracellular vesicles with non- and malignant colon epithelial cell lines

Malignant HCT116 cells were grown in McCoy’s medium and non-malignant 1459 colon cells were grown in EMEM medium seeded at a density of 1x10^5^ cells per plate. The number of EVs used for co-culture was normalized by using the NanoSight NS500 (NanoSight, Wiltshire United Kingdom) to count the total number of EVs within particular size 30-1000 nm. Each dish received 2 X 10^8^ EVs. 1459 cells were co-cultured with EVs from HCT116 cells and HCT cells were co-cultured with EVs from 1459 cells. Additionally, HCT116 cells and 1459 cells were co-cultured with EVs from both normal and malignant colon tissue. Cell lines were also co-cultured with their own isolated EVs for comparison. Co-cultures were maintained for 7 days in 5 % CO_2_ at 37 °C.

### Soft agar assay

Cells were harvested for soft agar cloning after a 7 days co-culture with EVs. Soft agar cloning was examined using 0.7 % agarose in PBS and mixed with 1× media with 15 % FBS. The top layer consisted of 0.35 % agarose in PBS, 1× media with 22.5 % FBS and 1 x 10^5^ cells per dish. Observations were made under the 40x objective and images of the plates were captured by a Nikon TE200 microscope.

Gray-scale Images (8 bit) were acquired with a Nikon TE200 inverted microscope (Nikon Inc. Melville NY) using a 10X Plan Fluor objective. Images were captured with a Spot RT3 digital camera (Diagnostic instruments, Sterling Heights MI) using the cameras built-in green filter to increase image contrast. iVision image analysis software (BioVision Technologies version 4.5.4, Exton, PA) was used to calculate area of the colonies. Images were calibrated so area measurements are expressed in micrometers.

### Migration assay

To assess migration, HCT116 control,1459 control, and HCT116 treated with 1459 EV (7 days) cells were plated in serum free medium in the upper chamber of a of an 8 μm pore Transwell filter (BD Bioscience, Bedford, MA) coated with Matrigel in a 24-well dish. Assays were set-up with 250,000 cells per condition. Cells were allowed to migrate at 37 °C, 5 % CO2 for 48 h, fixed with methanol and stained with 0.1 % w/v crystal violet. The filters were observed with 10X objective and migrating cells were determined in each well. Experiments were performed in triplicate and repeated twice.

### Protein extraction

Protein lysates from co-culture experiments and control cells were obtained after 7-day incubation using a ReadyPrep Sequential Extraction Kit (Bio-Rad). A ReadyPrep 2-D Clean-Up Kit (BioRad) was then used to combine and clean the sequential extractions. Total protein concentration was found using a BCA protein assay kit (Thermo Scientific). NuPAGE SDS-PAGE system (Invitrogen) (4-12 % acrylamide, Bis-Tris with MES SDS Running Buffer) was used to resolve the samples, and they were stained with Gel Code Blue Stain (Thermo Scientific). In order to reduce sample complexity, gel lanes corresponding to each sample were excised into 3 bands: high, medium and low molecular weight proteins. Each band was cut into 6 mm wide pieces before subjection to in-gel tryptic digestion. Post-digestion, the fractions were washed/dehydrated twice in a 1:1 solution of 1.0 M ammonium bicarbonate (Sigma) and 100 % ACN (Sigma). Disulfide bonds were reduced with 10 mM dithiothreitol (DTT) (Thermo)/0.1 ammonium bicarbonate for 45 min at 56 °C and alkylated with 55 mM iodoacetamide (IAA) (Sigma) for 30 min at room temperature, in the dark. Samples were washed/dehydrated twice, as explained above, and then digested by overnight with trypsin at 37 °C. Peptides were extracted, after trypsin digestion, using 25 mM ammonium bicarbonate and 100 % ACN, followed by two rounds of 5 % formic acid and 100 % ACN. Extracts were combined, dried using a vacuum centrifuge, and stored at -20 °C until LC/MS analysis.

### Liquid chromatography/ MS analysis of protein digests

Rhode Island Hospital COBRE Proteomics Core facility performed all mass spectrometry analysis by nano-LC-ESI-MS/MS using an Ultimate3000 nano-LC system (Dionex) controlled with Chromeleon software coupled to a QSTAR XL (Applied Biosystems, Concord, Ontario, CA) mass spectrometer. Tryptic digests were fractionated by reverse-phase chromatography using a C-18 PepMap 100 column (75 um id x 15 mm, 3um particle size, LC Packings/Dionex, Sunnyvale, CA) operating at a flow of 300 nL/min. Over a 40 min time period, a linear separation gradient was applied starting at 5 % (v/v) CAN in 0.1 % (v/v) formic acid (Buffer A) to 95 % (v/v) ACN in 0.1 % (v/v) formic acid (Buffer B). ESI was used to introduce the column elutate directly into the mass spectrometer.

Candidate ions were selected and fragmented using a standard information dependent acquisition (IDA) method. During MS/MS scans, one-second MS scans (range between 350 and 1800 Thompson, Thompson (Th) = Da/z) were used to identify candidates for fragmentation. MS/MS scans (2 s; range between 150 and 1800 Th) were collected up to three times after each survey scan. Candidates considered for fragmentation required an assigned charge in the range of +2 or +4.

### Data processing for protein identification and quantitation

Raw LC-MS/MS data was converted to mgf format using ABSciex MS Data converter software (v1.3 beta). Data in mgf format was used for protein identification with MASCOT v2.3.2 search engine (Matrix Science, Boston, MA, USA) by searching against a non-redundant human UniProt database (April 20^th^, 2012, containing 87, 656 protein entries). Parameters included the following: tryptic peptides with up to two missed cleavage sites, peptide tolerance of 0.2 Da, fragment tolerance of 0.5 Da, instrument type: ESI-QUAD-TOF, and variable modifications: methionine oxidation.

Raw files were converted to mzXML format using ABSciex MS data converter software (v1.3 beta) and uploaded along with MASCOT search results in .dat format into ProteoIQ software (v.2.3.08 BIOINQUIRE, Athens, GA, USA), for label-free protein quantitation and proteome comparisons. Spectral counting and relative intensity quantification were performed using precursor ion intensities. Parameters included the following: mass tolerance of 20 ppm, minimum peptide length of 6 amino acids, protein probability of 0.5, and peptide probability of 0.05. Proteins were further filtered using 0.9 protein probability and normalized according to the number of spectra in each sample.

### NF-κB luciferase reporter assay

Lipofectamine in serum-free medium was used to transiently transfect cells (3 x 10^5^ cells/60 mm dish) with 0.25 μg of a reporter plasmid containing NF-κB binding fragments with controls, as we have previously reported [16]. OptiMEM containing FBS was added to the cells after 3 h, at a final concentration of 20 %. Cells were harvested by scraping, washed twice with PBS and lysed in passive lysis buffer (Promega). Dual-Luciferase Reporter Assay (Promega) was used to evaluate the luciferase activity in the cytosolic supernatant in the presence of absence of the NF-κB inhibitor, BAY11-7082 (BAY). A luminometer (Lumat LB 9507, Berthold Technologies) was used to estimate transcriptional activity.

### Application of Small Interfering RNA (siRNA) against 14-3-3

HCT116 cells were plated in a 6-well plate, 24 h prior to transfection, in an antibiotic free growth medium. siRNA against 14-3-3 zeta, or equivalent amount of control siRNA solution, was mixed with a transfection reagent in OptiMEM and allowed to undergo complex formation for 30 min at room temperature. Cells were washed with OptiMEM and incubated for 6 h with the siRNA mix and OptiMEM. After 6 h, 1 ml of medium supplemented with 20 % FBS was added to the cells. Incubation was continued for a total of 48 h before harvesting. Western blot analysis was used to confirm inhibition of 14-3-3 zeta protein expression.

### Statistical methods

Cell culture experiments were all repeated a minimum of 3 times, unless otherwise indicated. Paired t-tests were used to determine statistical significance. P-values of 0.05, or less, were considered statistically significant.

## Results

### Extracellular vesicle-mediated induction of malignant colon cancer phenotype

**Fig. 1:**
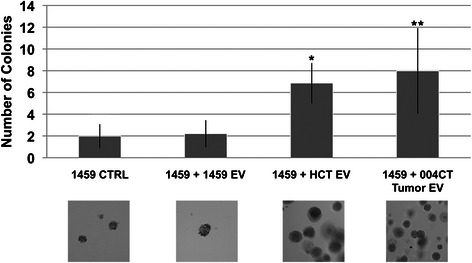
Extracellular vesicle-mediated induction of soft-agar growth. EVs were isolated from malignant (HCT116) cells and from patient tumor tissue (004CT Tumor). 1459 cells were co-cultured for 7 days with HCT116 EVs and 004CT Tumor EVs. In both experiments, cells were harvested and utilized for soft agar assay. 1459 cells were also co-cultured with EVs isolated from 1459 cells. Soft agar cloning was performed for 2 weeks and cell colonies were counted with 5 fields/dish using the 40× objective. There were 5 dishes/condition. To provide an estimation of colony size that was evaluated, the area for the colonies counted in an average field was determined. The average area (μm^2^) determined were: 1459 CTRL, 1988; 1459 + 1459EV, 2603; 1459 + HCT EV, 1860; 1459 + 004CT Tumor EV, 3841. The data represents the mean +/− s.d. of 2 independent experiments performed in triplicate. A paired t-test was performed to analyze the increase in soft agar colony formation of 1459 + HCT116 EVs when compared to untreated 1459 cells, *p < 0.00001. Increase in colony formation of 1459 + 004CT Tumor EV compared to untreated 1459 cells, **p < 0.00001, was also assessed

Anchorage independent growth is a major hallmark of malignant cells; therefore, we quantified EV-mediated phenotype changes in malignant colon cells using soft agar colony formation. EVs were harvested from a malignant human colon tumor cell line (HCT116) and from a patient tissue sample of malignant colon tumor (004CT Tumor). Two co-cultures were prepared with normal human colon fibroblast cell line 1459: 1459 + HCT116 EVs and 1459 + 004CT Tumor EVs. After a 7-day period of culture, each experimental condition was grown in soft agar to measure ability of anchorage independent growth. Malignancy includes increased anchorage-independent growth, so an increase in the number of colonies was viewed as a shift towards malignant phenotype. 1459 cells co-cultured with HCT116 EVs displayed a significant increase (p < 0.00001) in colony formation in comparison to the 1459 control cells (Fig. 1). The co-culture of 1459 and patient derived 004CT Tumor EVs also displayed a significant increase (p < 0.000001) in colony formation, suggesting an induction of malignant phenotype by EVs isolated from malignant colon tumor cells (Fig. 1). EVs from HCT116 cells were also co-cultured with HCT116 cells to determine if vesicles impact their own cells. There was no significant change in the number of colonies formed by the HCT116 + HCT116 EV co-culture, compared to HCT control (p = 0.33).

### Extracellular vesicle-mediated reversal of malignant colon cancer phenotype

**Fig. 2:**
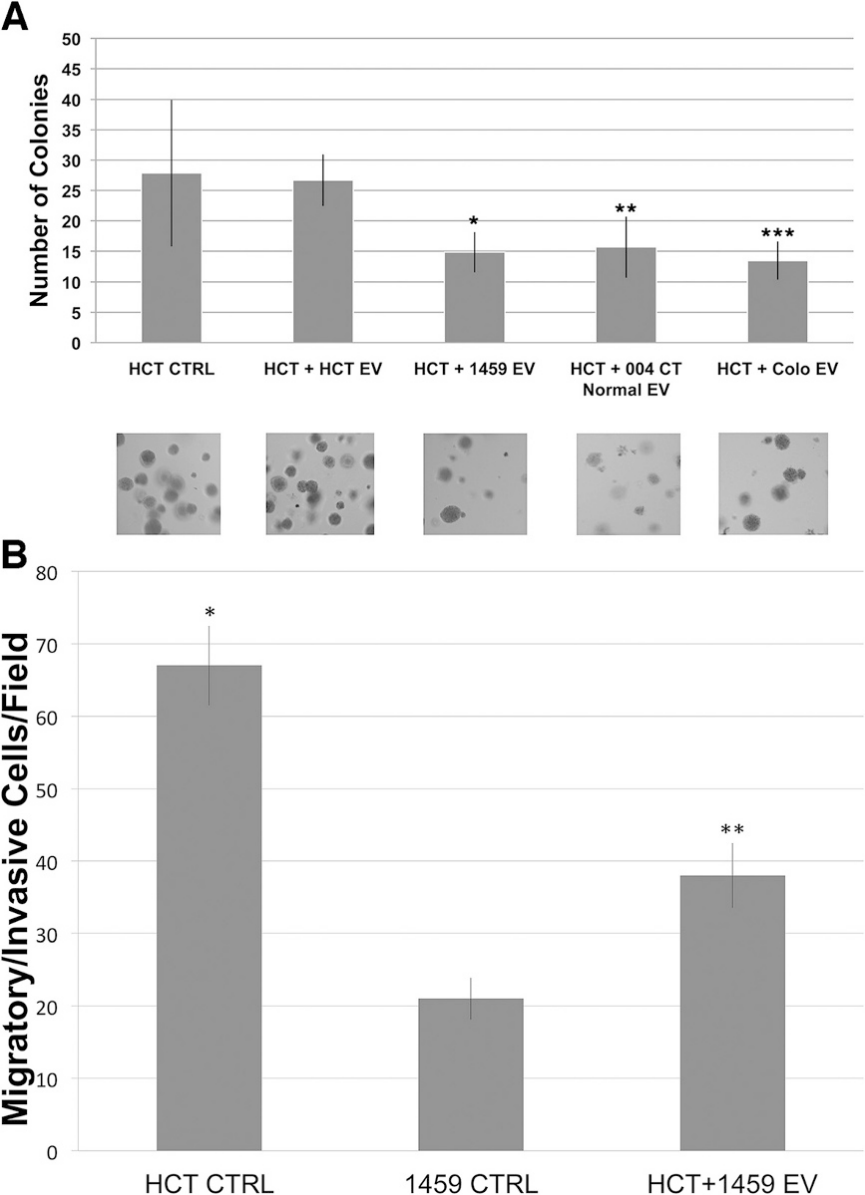
Extracellular vesicle-mediated reduction of soft-agar growth. **a** EVs were isolated from non-malignant (1459) cells. HCT116 cells were co-cultured for 7 days with 1459 EVs. EVs were also isolated from non-malignant patient tissue (004CT Normal and Colo) from 2 patients. HCT116 cells were co-cultured for 7 days with 004CT Normal EVs and Colo EVs, after which time soft agar was cloning was performed. An additional co-culture was set up with HCT116 cells and HCT116 EVs. The data represents the mean +/− s.d. of 2 independent experiments performed in triplicate. There were 5 dishes/condition. A paired t-test was performed to analyze the decrease in soft agar colony formation of HCT116 + 1459 EVs when compared to untreated HCT116 cells, * p < 0.00001. Decrease in colony formation of HCT116 + 004CT Normal EV compared to untreated HCT116 cells, **p < 0.00001, and HCT116 + Colo EV compared to untreated HCT116 cells, ***p < 0.00001, was also assessed. **b** Migration assay of HCT cells with and without EV treatment. Migration was measured by counting the number of cells on the filter after 48 h. An increase in migration of HCT116 cells relative to 1459 cells, *p < 0.0001, was analyzed using a paired t-test. Increased migration of 1459+ HCT EV compared to 1459 control cells, **p < 0.0001, was also assessed

EVs were harvested from a normal human colon fibroblast cell line (1459), as well as two patient tissue samples of normal colon epithelium (004CT Normal & Colo). Three different co-cultures were set up with malignant human colon tumor cell line HCT116, as follows: HCT116 + 1459 EV, HCT116+ 004CT Normal EV, HCT116 + Colo EV. The number of EVs was also normalized for these experiments (see above). After a 7-day incubation period, each experimental condition was grown in soft agar to measure ability of anchorage independent growth. A reduction in the number of colonies was viewed a shift towards a normal phenotype, reflecting the inability of anchorage-independent growth. The co-cultured cells were grown in soft agar for 14 days. HCT116 cells co-cultured with 1459 EVs displayed a significant decrease (p < 0.00001) in colony formation in comparison to the control HCT116 cells (Fig. 2a). A similar effect was seen from HCT116 cells co-cultured with patient tissue derived vesicles. Co-culture of HCT116 with 004CT Normal EVs and HCT116 with Colo EVs both displayed significant decreases (p < 0.00001, p < 0.00001) in colony formation (Fig. 2a). Our results suggest that EVs isolated from normal colon cells can mediate the reversal of malignant phenotype in colon cancer. An additional co-culture was set up with 1459 cells and EVs isolated from 1459 cells. There was no significant difference between the number of colonies formed by cells from 1459 + 1459 EV co-cultures and 1459 control cells (p = 0.28).

In addition to assessing the inhibition of HCT116 phenotype by 1459 EV treatment via soft agar assay, we performed transwell migration assay Our results indicate that co-culture of HCT116 cells with non-malignant 1459 EVs significantly inhibited cell migration (p < 0.0001) (Fig. 2b).

### Mass Spectrometry analysis of proteins lysates obtained from co-cultures

To further understand the mechanisms underlying the phenotype switch occurring after EV co-culture, mass spectrometry analysis was performed for protein identification. After the 7-day co-culture period, cells were harvested and used for soft agar cloning. The remainder of the cells were harvested and analyzed by mass spectroscopy. Table 1 shows a partial list of the proteins identified in 1459 cells co-cultured with HCT116 EV, proteins identified in 1459 cells co-cultured with 004CT Tumor EV, and 1459 cells co-cultured with 1459 EVs. The relative expression of each protein, as compared to 1459 control, is indicated as log2 relative expression. Also included in Table 1 are the log2 relative expressions of the partial list of proteins identified in 1459 cells co-cultured with HCT116 EV, as compared to HCT116 control.

**Table 1:** Comparison of some relative protein expression levels between 1459 co-cultured with malignant EVs a compared to 1459 control and HCT control

Accession #	Protein name	*log2 Relative Expression*			
		1459 v. 1459+ HCT EVs	1459 v. 1459 + 1459 EVs	1459+ 1459EV v. 1459+ 004CT(T) EVs	HCT v. 1459 + HCT EVs
P08648	Integrin Alpha-5	1.593	−1.631	3.063	0.23
P05556	Integrin Beta-1	0.779	−1.429	4.118	−0.228
P35232	Prohibitin	1.503		3.652	−2.135
P27348	14-3-3 theta	1.208	−4.004	3.66	1.436
P63104	14-3-3 zeta/delta	0.276	−3.643	2.827	1.032
P46940	Ras GTPase-activating-like protein	2.792	−2.328	0.755	0.976
P42224	STAT1	2.459			2.38

**Fig. 3:**
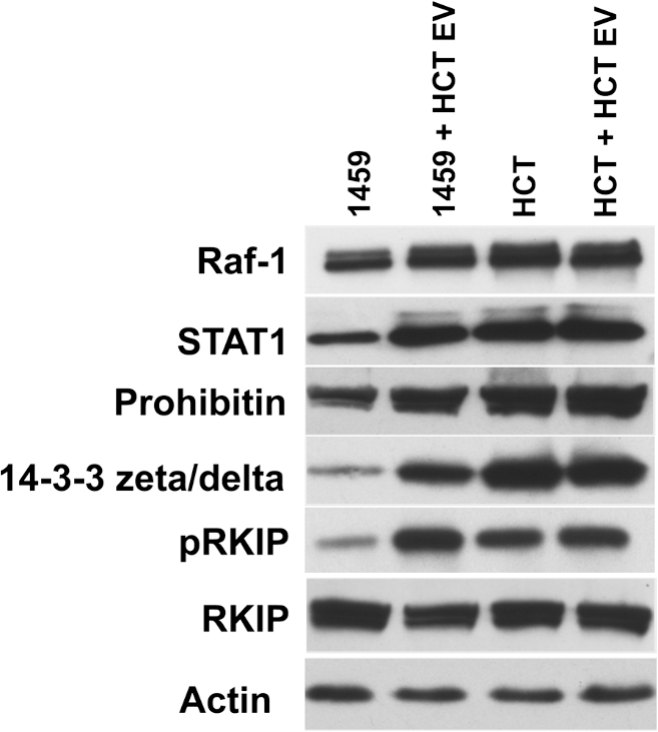
Extracellular vesicle mediated changes in cellular protein expression. EVs were isolated from malignant HCT116 cells. EVs were resuspended in PBS and co-cultured with non-malignant 1459 cells and malignant HCT116 cells. Upon completion of the 7-day co-culture, whole cell lysates were prepared for Western blot analysis, as reported. Western blot analysis results show and increased expression of Raf-1, pRKIP, STAT1, Prohibitin and 14-3-3 zeta/delta in 1459 + HCT116 EV co-cultures, as compared to 1459 control. A slight decrease expression of RKIP is also shown in this comparison. We examined these proteins based on LC-MS data (Table 1)

Many of the isoforms in the 14-3-3 family of proteins play key roles in promotion of a malignant phenotype [17–19]. Our mass spectroscopy analysis revealed an increased expression of 14-3-3 zeta/delta in co-cultures of 1459 cells with 004CT Tumor and HCT116 EVs, which was confirmed with Western blot analysis (Fig. 3). Further, a decrease in 14-3-3 zeta/delta was observed in 1459 cells cultured with 1459 vesicles. This further supports the notion that 14-3-3 is a key protein in imparting the malignant phenotype of colon tumor cells.

Prohibitin and IGQAP1 were also identified by MS as having similar expression patterns as the 14-3-3 proteins. Increased expression of prohibitin and Raf-1 in 1459 co-cultures with malignant vesicles (HCT116 and 004CT Tumor) was validated with Western blotting (Fig. 3). LC-MS/MS detected up-regulation of IGQAP1, or Ras GTPase-activating-like protein, which we used as basis to explore Raf-1 expression, due to recent research implicating IGQAP1 as a key scaffold protein for the activation of ERK1/2 [20]. Raf-1 is a major upstream regulator of ERK1/2, two key proteins in the RAF-MEK-ERK pathway controlling cell fate, including differentiation, apoptosis and proliferation [21, 22]. Raf-1 function is linked to increased levels of pRKIP [23], and Western blotting revealed an increase in pRKIP expression in 1459 + HCT116 EV co-culture (Fig. 3). Prohibitin is seen to be expressed at higher levels in many types of cancer and is said to play a role in cell cycle regulation, apoptosis and cellular senescence [24, 25]. Of important note is a decrease in prohibitin expression of 1459 cells co-cultured with HCT116 EVs in comparison to control HCT116 cells. Prohibitin was also not detected by MS in the 1459 + 1459 EV co-culture. Again, similar to 14-3-3, IGQAP1 was detected by MS to have a decreased expression in 1459 + 1459 EVs relative to 1459 control cells.

Signal Transducer and Activator of Transcription 1 (STAT1) is a member of the STAT family of transcription factors. In the past, STAT1 was seen as a tumor suppressor, however, recent research suggests that up-regulation of STAT1 can promote immunosuppression in the tumor microenvironment causing a more favorable environment for tumor growth [26, 27]. Mass spectrometry revealed an up regulation of STAT1 in 1459 co-cultured with HCT116 EVs, relative to both the 1459 and HCT116 control cells. Western blot analysis confirmed these results (Fig. 3).

### Co-culture with malignant extracellular-vesicles increases transcription of NF-κB

**Fig. 4:**
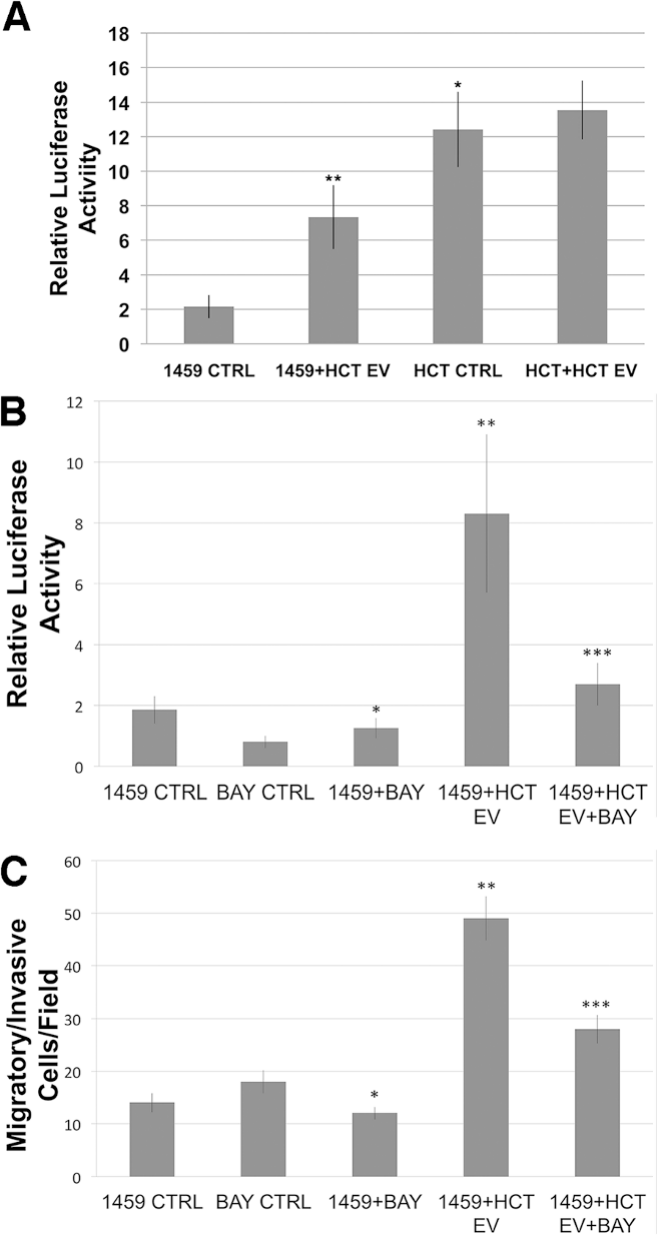
Extracellular vesicle induced transcription of NF-κB. **a** EVs were isolated from HCT116 cells. EVs were resuspended in PBS and co-cultured with non-malignant 1459 cells and malignant HCT116 cells. After 7 days of co-culture, cells were transfected with NF-κB reporter plasmid to measure NF-κB transcriptional activation [16]. After 24 h, the samples were harvested, washed twice, lysed and combined with a luciferase assay reporter. The data is reported as the mean +/- s.d. of 2 independent experiments performed in triplicate. Relative to 1459, HCT116 cells showed increased activity of NF-κB transcription, *p < 0.00001. Increased relative activity of NF-κB transcription was also seen in 1459 cells co-cultured with HCT116EVs, relative to 1459 control, **p = 0.00004. **b** EVs were isolated from HCT116 cells and NF-κB transcriptional reporterassay was performed as described in **a** in the presence or absence of 3 μm BAY, a irreversible inhibitor of NF-κB [30]. Relative to 1459 control, 1459 cells in the presence of 3 μm BAY showed decreased activity of NF-κB transcription, *p = 0.025. Increased relative activity of NF-κB transcription was observed in 1459 cells co-cultured with HCT116EVs, relative to 1459 control, **p < 0.0001. In the presence of 3 μm BAY, 1459 + HCT116 EV cells showed decreased activity of NF-κB transcription, as compared to those in the absence of BAY, ***p < 0.0001. **c** Cell migration assay was performed using 1459 cells co-cultured with HCT116 EV in the presence or absence of 3 μm BAY [30]. Relative to 1459 control, 1459 + BAY cells showed decreased cellular migration, *p = 0.047, as assessed by paired t-test. An increase in cellular migration was assessed in 1459+ HCT116 EV co-cultured cells, relative to 1459 control, **p < 0.0001. Co-culture of 1459 cells with EVs from HCT116 cells treated with 3 μM BAY showed decreased cellular migration, ***p < 0.0001, compared to 1459 + HCT116 EV co-cultured cells. The assay was performed twice in triplicate

NF-κB signaling controls DNA transcriptions and cellular responses to stress, cytokines and both bacterial and viral antigens. In particular, NF-κB has been implicated in both anti-apoptotic and inflammatory processes in the development and progression of CRC [28, 29]. After a 7-day co-culture, HCT116, HCT116 + HCT EV, 1459 and 1459 + HCT EV cells were harvested and transfected with a reporter plasmid to measure NF-κB activation, as we have previously reported [16]. Forty eight hours after transfection, cells were harvested, washed twice, lysed and combined with a luciferase assay reporter. Relative to 1459 control, 1459 cells co-cultured with malignant EVs showed a significant increase (p = 0.00004) in NF-κB transcription activity. A significant increase (p < 0.00001) in NF-κB transcription was also seen in HCT116 cells relative to 1459 cells (Fig. 4). No changes in NF-κB transcription were seen between HCT116 cells and HCT116 co-cultured with HCT EVs (p = 0.21). We examined the localization of p65 before and after HCT116 EV treatment and determined that p65 remained in the cytosol (Data not shown). This indicates that another component of HCT116 EV cargo enhanced NF-κB transcription. These findings support the role of NF-κB in the promotion of CRC and suggest NF-κB as a target in EV-mediated promotion of malignant phenotype.

Our results suggest the NF-κB activation in 1459 cells via co-culture with HCT116 EVs promotes phenotype shifting. To explore the role of NF-κB in phenotype shifting, we performed NF-κB luciferase and migration assays in the presence of BAY117082 (BAY), a known inhibitor of NF-κB activation in HCT116 cells [30]. 1459 NF-κB luciferase transcription reporter activation and cell migration were significantly reduced (p < 0.0001), respectively for both, in cells treated with BAY and HCT116 EV (Fig. 4b, c).

### siRNA repression of 14-3-3 zeta reduces extracellular-vesicle mediated induction of malignant colon cancer phenotype

**Fig. 5:**
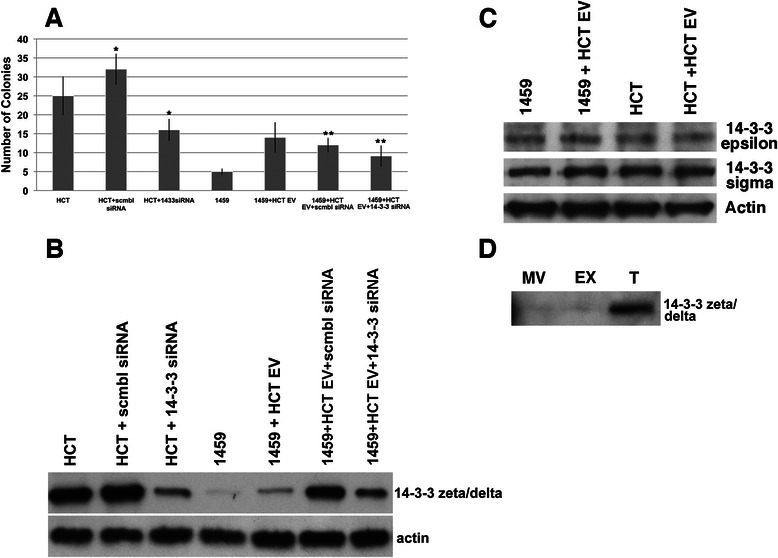
siRNA mediated Knock Down of 14-3-3 zeta/delta reduces soft agar colony formation. **a** HCT116 cells were transfected with siRNA to 14-3-3 zeta/delta, or equivalent amount of control siRNA solution. After 48 h incubation with siRNA to 14-3-3 zeta/delta, or equivalent amount of control, HCT116 cells were harvested for soft agar cloning and saved for Western Blot analysis. EVs were harvested from the HCT116 cells transfected with siRNA to 14-3-3 zeta/delta. EVs were resuspended in PBS and co-cultured with 1459 cells. After 7 days of co-culture, soft agar coloning was performed. There were 5 dishes/condition. The data represents the mean +/− s.d. of 3 independent experiments performed in quadruplet. A paired t-test was performed to analyze the decrease in soft agar colony formation of HCT116 transfected with 14-3-3 zeta/delta siRNA, compared to HCT116 transfected with scrambled siRNA as control, *p < 0.00001. Decrease in colony formation of 1459 + HCT116 14-3-3siRNA EV when compared to 1459 + HCT116 scramble siRNA EV was assessed, ** p = 0.0039. **b** After co-culture, cells were harvested and a portion of the cells were prepared as whole cell lysates for Western blot analysis. Western blot analysis confirmed the reduced expression of 14-3-3 zeta/delta in HCT116 + 14-3-3siRNA compared to HCT116 and HCT116 + scramble siRNA controls. Reduced expression of 14-3-3 zeta/delta was also seen in the co-culture of 1459 + HCT116 14-3-3 siRNA EV as compared to 1459 + HCT116 EV and 1459 + HCT116 scramble siRNA EV controls. **c** Western blot analysis of 14-3-3 sigma and epsilon protein levels of 1459 cells after co-culture with HCT116 EVs. **d** Western blot of 14-3-3 zeta/delta protein from vesicles isolated from 3 differential centrifugation preparations. MV = microvesicle fraction; EX = exosome fraction; T = MV and EX fraction

Repression of 14-3-3 zeta via siRNA knock-down in HCT116 cells was used to observe the putative role of 14-3-3 zeta in EV-mediated promotion of a malignant phenotype in non-malignant colon epithelial cells. HCT116 cells were transfected with 14-3-3 zeta siRNA or scramble siRNA for 48 h. After transfection, the cells were grown for 7 days before harvesting. Media was collected for EV isolation and cells were harvested for protein lysates and soft agar assay. Western blot analysis confirmed reduced expression of 14-3-3 in HCT116 cells transfected with siRNA to 14-3-3 zeta, as compared to both HCT116 and HCT116 + scrambled siRNA controls (Fig. 5b). Non-malignant 1459 cells were co-cultured with each of the three types of vesicles for a period of 7 days. After 7 days, cells were harvested for protein lysates and soft agar assay. HCT116 cells with a reduced expression of 14-3-3 zeta showed a significant decrease in colony formation, compared to HCT116 cells transfected with control scramble siRNA (*p* < 0.00001) (Fig. 5a). Additionally, the co-culture of 1459 and vesicles isolated from HCT116 + 14-3-3 siRNA cells showed a significant decrease in colony formation, compared to co-cultures with vesicles isolated from HCT116 + scramble siRNA control (*p* = 0.0078) (Fig. 5a). Western blot analysis confirmed the decrease in endogenous 14-3-3 zeta levels (Fig. 5b). These results indicate that 14-3-3 zeta may be responsible, in part, for conferring the malignant phenotype via EVs in HCT116 cells.

We examined two other isoforms of 14-3-3 proteins, sigma and epsilon, to determine if our affect on phenotype shifting was 14-3-3 zeta/delta-specific. Western blot analysis indicated no change in the protein levels of 14-3-3 sigma or epsilon after co-culture of 1459 cells with HCT116 EV (Fig. 5c).

Lysates were prepared from HCT116 EVs after differential centrifugation to isolate the microvesicle, exosome and total (microvesicle + exosome) fractions. Western blot analysis indicated that 14-3-3 zeta/delta was present in the total EV cargo content prior to co-culture experiments (Fig. 5d).

## Discussion

A more complete understanding of the tumor microenvironment is critical in understanding underlying mechanisms of metastatic disease. Abundant in this environment are membrane bound EVs, which are secreted at increased rates in cells exhibiting a cancerous phenotype [10]. Through fusion with neighboring cells, EVs act as mediators of horizontal protein and/or gene transfer. Recent research has implicated this and other mechanisms resulting in the exchange of genetic material between non-parent and donor cells, in tumor progression. Focus has been placed on cell-cell interactions including cellular fusion and cellular response to both plasmids and viral particles [31]. There are, however, other important factors secreted by cells. *Ogata-Kawata, et. al*. recently identified seven micro-RNA found to be overexpressed in serum samples from patients with CRC, providing evidence that secreted factors might play a role in the regulation of gene expression [32]. Similarly, *Kruger, et a*l, described differential expression of miRNA in EVs isolated from two breast cancer cell lines [33]. In another study, an induction of phenotypic changes in prostate cancer cells through EV exposure was detected. They also observed EV-mediated reversal of chemosensitivity in otherwise resistant prostate cancer cell lines [34]. It is highly plausible that EVs can traffic microRNA, which in turn mediates relevant changes in protein expression. In our study, we focus on the role of EVs in intracellular communication and identify their capacity to induce phenotypic changes in colon cancer cells. Observation of these changes supports the role of EVs in the horizontal transfer of proteins and provides targets for unique CRC therapies.

In this manuscript, we show that EVs can mediate the transfer of both malignant and non-malignant cellular phenotype in colon cells. EVs isolated from established human colon cell lines and fresh patient tumor samples were both shown to mediate changes in cellular phenotype, supporting the concept that these vesicles transfer and/or induce biological components that enter the cells and enhance or minimize aspects of cellular function related to malignancy. *In vitro*, we assessed malignant phenotype through soft agar colony formation as quantification of anchorage independent growth. Significantly increased colony formation after co-culture of normal colon cells with malignant-derived vesicles (Fig. 1), and decreased colony formation and cell migration after co-culture of malignant colon cancer cells with non-malignant-derived vesicles provides evidence for phenotypic change (Fig. 2).

Along with identifying the ability of EVs to reverse and promote malignancy in CRC, a major aim of this study to was identify putative protein targets for novel therapies. We used proteomic analysis to assess levels of protein expression as a means of observing genetic changes resulting from EV-mediated horizontal gene transfer. Mass spectrometry analysis on samples of cells before and after co-culture revealed significant alterations in expression of key proteins, indicating that either genetic material encoding proteins, or the proteins themselves, are being released in EVs secreted by cells. Observation of EV-mediated reversal of malignant phenotype in colon cancer cells suggests EVs as a potential therapy for CRC. After evidence of phenotype switching, we chose to further explore the protein changes mediating the induction of malignancy, with the goal of further understanding the intracellular changes promoting an inherently non-malignant colon epithelial cell to transform into one that exhibits a malignant phenotype. Given that EVs have been found in serum, it is possible that EVs released by localized colon tumor cells can travel in the bloodstream and help to promote metastasis [35, 36].

Our data suggests STAT1 is affected by the horizontal transfer of genetic material, as mediated by EVs. STAT1 belongs to the family of STAT proteins that mediate the transcription of various target genes by localizing to the nucleus and binding to gene promoters, following activation by interferon-gamma [37]. We have shown that another member of the STAT family, STAT3, has been implicated in poor prognosis of CRC [38]. Conversely, one of the largest arguments for STAT1 in cancer progression has been the reverse: a pro-apoptotic protein acting as a tumor suppressor [39, 40]. The findings of this study support another hypothesis that STAT1 behaves as an oncogenic protein. A small section of recent research supports this argument. *Hix et al* identified a relationship between increased expression of STAT1 and breast cancer growth, attributed in part of the resulting over expression of pro-inflammatory cytokines [41]. These cytokines, including Interleukin-6 (IL-6), have been shown to foster a microenvironment conducive to the progression of various types of cancer [42–44]. STAT1 up-regulation was seen in the co-culture experiments, supporting recent research suggesting an oncogenic role of STAT1 in the tumor microenvironment. Future projects should aim to further understand the oncogenic role of STAT1 and it’s validity as a drug target.

Prohibitin’s potential as an oncogenic protein is heavily debated, given that it was first identified in tumor suppression through enhanced p53 transcription [25, 45, 46]. *Sievers et al* recently identified a decrease in proliferation of various cancer cell lines after silencing of prohibitin [25]. In another recent study, *Panagopolous et al* identified enhanced expression of prohibitin in EVs isolated from patient prostate tumor samples [34]. Our research further supports the growing evidence implicating prohibitin in tumor progression, given that our findings demonstrating that 1459 cells acquire the ability to grow significantly in soft agar when co-cultured with malignant EVs (Fig. 1). These malignant EVs likely mediate a transfer of biomaterials causing an induction or direct transfer of prohibitin as a mechanism of the non-malignant to malignant transformation observed in our model.

The major pathway affected by changes in prohibitin levels is the RAF-MEK-ERK pathway, which serves as a major mediator of a variety of signaling pathways controlling the survival of cells, as well as differentiation and cell-cycle regulation [47]. IQGAP1, or Ras GTPase-activating-like protein, has also been identified as an important regulator of the RAF-MEK-ERK pathway, specifically with increased expression of IQGAP1 leading to increased phosphorylation of ERK1/2 [21, 47, 48]. Analysis of LC-MS/MS data indicated an increased expression of this protein in co-cultures of 1459 with malignant EVs. Based upon our proteomics findings, we further explored the expression of Raf-1 and its regulatory proteins. Raf-1 is an upstream regulator of the RAF-MEK-ERK pathway, recruited by Ras in response to growth factor and/or cytokine signaling. Activation of Raf-1, through phosphorylation, in-turn leads to a phosphorylation cascade of MAPK kinases that compose the RAF-MEK-ERK dependent regulation of cell processes [49, 50].

RKIP is known to interfere with function of Raf-1 by preventing its ability to then phosphorylate MEK to continue the signaling cascade. More specifically, Raf-1 is bound by RKIP at the phosphorylated N-terminal, which blocks the binding of MEK [51]. Binding between RKIP and MEK has also been seen, again, blocking the signal cascade [52]. Decreased expression of RKIP by microRNA224 has previously been implicated in metastasis of breast cancer [53]. MicroRNA, however, is just one mechanism responsible for reduced RKIP expression in cells. Phosphorylation of RKIP by protein kinase C (PKC) at the Ser153 residue leads to inactivation, causing the release of Raf-1 and subsequent reestablishment of RAF-MEK-ERK mediated proliferation [24]. Studies of CRC patient tumor samples by *Minoo,et al* show an association between poor prognosis and reduced or lost expression of RKIP [54]. *Cross-Knorr et al* shows a similar association, recognizing an increased expression of pRKIP in CRC patients with poor prognosis [38]. Western blot analysis of our co-culture samples showed an increased expression of Raf-1 and pRKIP in 1459 + HCT EV compared to 1459 controls (Fig. 3), supporting these findings and further implicating the RAF-MEK-ERK pathway in the induction of malignancy by EVs.

LC-MS/MS data indicated IQGAP1 was expressed at a significantly lower level after 1459 co-culture with its own non-malignant vesicles, and prohibitin was undetectable by mass spectrometry in the same condition. Given the reduction in expression, it is plausible that the 1459 EVs carry miRNA acting to down regulate key proteins in RAF-MEK-ERK mediated signaling. This indicates the potential that EVs secreted from non-malignant and malignant cells affect the same pathways in reciprocal fashion. Vesicles isolated from non-malignant colon cells and malignant colon cells should be further assessed for genetic profiles, including miRNA content, for a better understanding of the mechanism behind EV induced mediation of key protein levels.

In addition to inhibiting the RAF-MEK-ERK cascade, RKIP is also known to have an inhibitory effect on the NF-κB pathway through upstream interactions with its activating kinases [17, 55]. NF-κB is established to play key roles in both anti-apoptotic processes of the cell, as well as promotion of inflammation. Enhanced expression of NF-κB transcription factors have been linked to a variety of human carcinomas, including CRC tumors [56, 57]. NF-κB is activated through the degradation of its inhibitory proteins, leading to release of the NF-kB dimer and it’s translocation to the nucleus [58]. NF-κB then targets specific DNA sequences to facilitate gene expression, specifically of genes critical in cellular death. Normally, tumor necrosis factor (TNF) would initiate a signaling cascade through the binding of caspase-8 and FADD, leading to apoptosis in the cell [59]. However, increased levels of TRAF1 and TRAF2, as a result of NF-κB expression, were enough to block this caspase-8 activation and inhibit cell death [60].

Additionally, a significant increase in NF-κB expression is seen in both patients with inflammatory bowel disease, as well as *Helicobacter pylori* induced gastritis [61]. This increase in NF-κB is coupled with increased levels of pro-inflammatory cytokines including e-selectin and Interleukin-8 [62]. An inflammatory environment has been implicated in tumor progression. In our experiment, luciferase reporter assay of 1459 + HCT116 EV co-culture revealed increased transcriptional activity of NF-κB, relative to 1459 control (Fig. 4a). HCT116 EV-mediated 1459 NF-κB activation and cell migration were abrogated in the presence of the irreversible NF-κB inhibitor BAY (Fig. 4b,c). Thus, our findings suggest that malignant EVs induce expression of NF-κB as a means of suppressing apoptosis in HCT116 cells and promoting an inflammatory microenvironment that aids in the transformation to a malignant phenotype.

We observe enhanced levels of 14-3-3 zeta/delta in 1459 cells following co-culture with HCT116 and patient derived malignant EVs through both proteomic and Western blot analysis. (Table 1, Fig. 3a). The 14-3-3 family of seven distinct proteins serves a diverse role in cellular function through binding with kinases, phosphatases and transmembrane receptors to regulate intracellular signaling, cell cycle control, apoptosis and proliferation [18]. Of the 14-3-3 proteins, 14-3-3 sigma has been implicated in both tumor suppression through regulation by p53, and tumor progression. While initial findings supported 14-3-3 sigma-related cell-cycle arrest, recent publications provide contradictory evidence which suggests increased expression of 14-3-3 sigma in cancer cells [63]. Knock-down of 14-3-3 sigma decreased resistance to cisplatin in CRC, suggesting pro-apoptotic function of 14-3-3 sigma [64]. Similarly, other members of the 14-3-3 family have been identified as up-regulated in multiple cancers [19, 65]. 14-3-3 zeta/delta has been specifically been linked to regulation of Raf function through accompaniment to the cellular membrane [66].

Further investigation into the role of 14-3-3 zeta/delta in EV-mediated transformation of colon cells was completed through a reduction of protein expression using siRNA. 1459 cells co-cultured with EVs harvested from HCT + 14-3-3 siRNA cells showed reduced soft agar growth, relative to co-cultures with control HCT + scramble siRNA EVs (Fig. 5a). This finding, coupled with decreased soft agar colony formation in HCT + 14-3-3 siRNA cells compared to HCT + scramble siRNA controls (Fig. 5a), supports the hypothesis that 14-3-3 zeta/delta is responsible, in part, for EV-mediated transformation. Cells expressing lower levels of 14-3-3 zeta/delta see a reduced capacity for 14-3-3 secretion in EVs, and are therefore limited in their ability to induce phenotypic changes in neighboring cells. Our results do not indicate any alterations in 14-3-3 sigma or epsilon protein levels after co-culture with HCT116 EVs (Fig. 5c). This indicates, again, that 14-3-3 zeta/delta is likely responsible for the phenotypic changes we observe, which is corroborated by the presence of 14-3-3 zeta/delta in the cargo of HCT116 EV (Fig. 5d).

Along with promoting tumorigenesis through regulation of the RAF-MEK-ERK pathway, 14-3-3 proteins have also been implicated in the binding of pro-apoptotic protein BAD (BCL-2 antagonist of cell death) [20]. The BCL-2 family of proteins are key regulators of apoptosis through control of mitochondrial outer membrane permeabilization (MOMP). In the mitochondria, BAD mediates the formation of a heterodimer between anti-apoptotic proteins BCL-2 and BCL-X_L_, thus preventing their inhibition of BAX (BCL-2-associated X protein) [67]. BAX is a key protein required to form mitochondrial pores allowing mitochondrial proteins, including cytochrome c, to activate caspases in the cytosol, resulting in apoptosis [68, 69]. We observe increased expression of 14-3-3 in cells expressing malignant phenotype (Fig. 3), providing further evidence that 14-3-3 is crucial in cell survival through binding of BAD to prevent its relocation to the mitochondria. Given their dual role in the survival and proliferation of cells, 14-3-3 proteins may serve as a key targets in therapies for CRC.

**Fig. 6:**
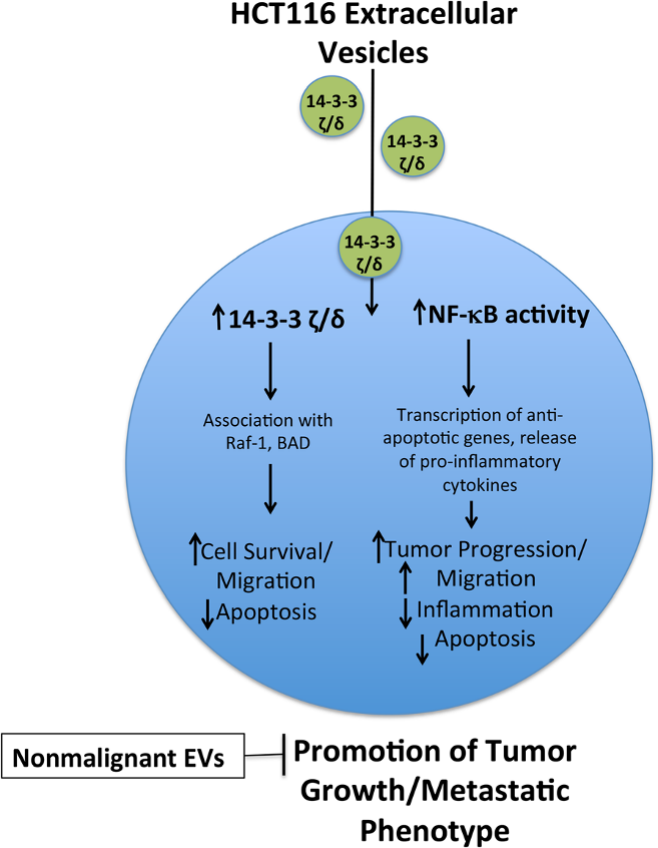
Schematic representing our model indicating the transfer of 14-3-3 zeta/delta (circles) by HCT116 EVs with the activation of NF-κB with downstream signaling events regulated by both 14-3-3 zeta/delta and NF-κB leading to the malignant phenotype that is inhibited by non-malignant EVs

Collectively, our results indicate that HCT116 EVs carry 14-3-3 zeta/delta as part of their cargo that is transferred to recipient cells after co-culture (Fig. 6). This also results in the activation of NF-κB (Fig. 6). Consequently, there is a promotion of a series of events leading to HCT116 cancer cell survival, which can be abrogated, in part, by co-culture with non-malignant EVs (Fig. 6).

## Conclusion

In summary, this study identifies EVs as an important mediator of horizontal transfer of genetic material in the promotion of a malignant phenotype with an increased expression of key RAF-MEK-ERK pathway mediating proteins including 14-3-3 zeta/delta, Raf-1, pRKIP and NF-κB. These proteins may serve as key targets for novel therapies in patients with CRC tumors. Additionally, EV-mediated reversal of malignant phenotype was observed in this study, suggesting the use of EVs as tumor biomarkers or as a therapeutic agent. Analysis of the contents of both malignant and non-malignant CRC EVs derived from patient samples, in the future, would provide additional data for the assessment of EVs as therapeutic agent or for biomarkers (i.e., 14-3-3 and prohibitin) for potential therapeutic targets and drug development for CRC.

## Abbreviations

EV: Extracellular vesicle
CRC: Colorectal cancer
KRAS: Kirsten rat sarcoma viral oncogene homolog, EGFR, Epidermal growth factor receptor
PTEN: Phosphatase and tensin homolog
BRAF: v-Raf murine sarcoma viral oncogene homolog B1
PI3KCA: Phosphatidylinositol 3-kinase
RKIP: Raf Kinase Inhibitor Protein
STAT1: Signal transducer and activator of transcription 1
NF-κB: Nuclear factor kappa-light-chain-enhancer of activated B cells
LC-MS: Liquid chromatography-mass spectrometry
IL-6: Interleukin-6
siRNA: Small interfering RNA
BAY: BAY117082
